# Tax evasion, psychological egoism, and revenue collection performance: Evidence from Amhara region, Ethiopia

**DOI:** 10.3389/fpsyg.2023.1045537

**Published:** 2023-02-08

**Authors:** Renyan Mu, Nigatu Mengesha Fentaw, Lu Zhang

**Affiliations:** School of Management, Wuhan University of Technology, Wuhan, China

**Keywords:** tax evasion, psychological egoism, tax education, technology, tax revenue collection performance

## Abstract

Tax evasion is the illegal withholding or underpayment of taxes, typically accomplished by intentionally providing false or no evidence to tax authorities. Tax evasion has had a severe detrimental influence on the economy of the Amhara National Regional State, Ethiopia. The Amhara Regional State lost tax revenue in recent years due to tax evasion. The objective of this study was to see how tax evasion, taxpayers’ psychological egoism, and other relevant factors affect tax revenue collection performance in the Amhara Region, Ethiopia. Data were collected from 395 VAT-registered taxpayers through a structured questionnaire. The structural equation model and multiple regression analysis method were utilized for empirical test based on the softwares of SPSS and AMOS. This research revealed that tax evasion and psychological egoism negatively affect tax revenue collection performance. Tax education and technology significantly and positively affected tax revenue collection performance. Meanwhile, the relationships between the above factors (tax evasion, tax education, and technology) and the tax revenue collection performance are reliably mediated by taxpayers’ psychological egoism. Those findings can give clues to researchers, tax experts, and policymakers for improving the tax revenue collection performance in Amhara Region. The government can enhance public education to reduce tax evasion and such misbehavior caused by taxpayers’ psychological egoism. Meanwhile, the most up-to-date tax invoicing technologies, like artificial intelligence and machine learning technology should be adopted.

## 1. Introduction

Taxation is a mandatory expense or transfer of resources from the individual to the public sector paid based on criteria and without regard to specific advantages received to achieve some of the country’s economic and social goals (Olaoye et al., 2018; Faheem and Alzoubi, 2019; Tarfa et al., 2020). On top of this, taxation is a public duty and a contribution levied by the government on its subjects and businesses to empower them to finance or operate public facilities and fulfill other social responsibilities. As a result, taxes are the primary foundation of government revenue. But there are different problems in collecting appropriate taxes. Most researchers agreed that taxation is compulsory payment by taxpayers to public investments.

On the other hand, many researchers pointed out that tax evasion and tax avoidance are the most serious problems faced by Africa in terms of taxation. Tax evasion is the illegal behavior of taxpayers who fail to pay or underpay taxes in violation of the provisions of the tax law. Tax avoidance is the dynamic tactic by which a taxpayer attempts to reduce or eliminate his tax liability without actually breaking the law (Badara, 2012; Olsen et al., 2019). Such misbehavior has produced a massive gap between actual and projected revenue. Ilham and Hayon (2019) and Quick (2020) argue that if people can avoid paying the tax to which they should logically be subject within the general scope of the tax, the theoretical justice of the tax is lost to a great extent. Tax evasion and avoidance undoubtedly deprive the tax revenue of the government, resulting in a discrepancy between prospective and actual tax collections. This research aims to close that gap.

In the Amhara Region, the average performance of amount of tax collection in a million Ethiopian birr from 2009 to 2018 was 98,102.63; the 10-year tax collection document shows that the difference between the minimum value of 23,583.26 in 2009 and the maximum value of 176,102.82 in 2018, the amount of tax collection is increasing (Nigatu, 2022). However, the potential to collect enough tax and cover the communities’ infrastructure development needs is very high. The critical problems are tax evasion, taxpayers’ psychological egoism, and related issues. Although tax evasion and avoidance are the main challenges of every tax system, the Amhara Region situation appears to be unique in the current situation in Ethiopia. The collection of taxes from the self-employed, such as business taxpayers, contractors, and different professional taxpayers related to their fields, such as lawyers, tax accountants, and architects, is a severe problem under Amhara Region’s direct taxation system.

Most taxpayers in Amhara Region reported under-invoicing, exaggerated expenses, and fictitious supporting documents to evade taxable incomes; all these practices can emanate from taxpayers’ psychological egoism. Most taxpayers are self-centered and waiting for obligatory government actions. Tax evaders deliberately refuse to pay taxes by declaring annual losses (Barbu et al., 2022; Siglé et al., 2022). Taxpayers live and enjoy lifestyles contradictory to their stated income, which sometimes exposes their businesses to how they are effective and profitable. Meanwhile, tax accountants who serve taxpayers sometimes encourage taxpayers to evade their taxable incomes in Amhara Region, Ethiopia. Taxpayers’ accountants lead taxpayers to use tax loopholes like tax avoidance; they also stand at the side of taxpayers’ benefits and reduce tax liability. As a result, many taxpayers soundly VAT-registered taxpayers evade their taxable incomes in Amhara Region. In addition, due to taxpayers’ psychological egoism, the hidden illegal gains from tax evasion and the luxurious lives of egoist taxpayers might influence compliant taxpayers and push them to evade their taxes, which affects the revenue collection performance in Amhara Region. From this perspective, taxpayers’ psychological egoism may serve as a crucial mediator between tax evasion and revenue collection performance.

Moreover, the teaching methodology on tax education in the Amhara Region is not smart and not in line with the situation; community leaders don’t actively organize tax education activities, and the regional conflict terminates house-to-house education. The existing technology has been served for the last 15 years and does not include all tax types like auditing. Auditors audit all taxpayers’ transactions manually except for receiving transaction reports. Inefficient tax education practices and longtime served (outdated) technology affected the Amhara Region tax revenue collection performance.

Given the above aspects, this research combined tax evasion, taxpayers’ psychological egoism, and other possible influencing factors (tax education and technology) with tax revenue collection performance, investigated their impacts on tax revenue collection performance in the Amhara Region, and analyzed the potential mediating influencing mechanism of taxpayers’ psychological egoism. The findings could not only make up for the existing research blank but also give empirical references for the authorities in Amhara Region to take effective measures to improve their tax revenue collection performance.

## 2. Literature review

The literature part of this research presents the theory of psychological egoism and the theory of tax evasion to show the generally accepted application of the two ideas. The next part explains the empirical reviews based on the required variables. Finally, the independent, the mediating, and the dependent variable measuring instruments with the supporting literature are described in different ways. The relationship between the independent, the mediating, and the dependent variables with the proposed hypotheses is presented with supporting references. It may be helpful to the research areas to take remedial action on the issues and valuable to readers and researchers to cross-check and use for further research. Thus, the theoretical and empirical review parts are as follows.

### 2.1. Theoretical review

#### 2.1.1. Theory of psychological egoism

The first theory about the nature of human motives is called psychological egoism. It holds that everyone’s behavior, activities, and decisions are driven by self-interest. Psychological egoism is reductive because it contains all ultimately selfish motivations (Feinberg, 2018; Tomaszewski, 2021).

According to the theory of psychological egoism, individuals with self-interest do not worry about social and economic capital that can benefit society. The theory of psychological egoism that each person is psychologically wired to only look out for himself or his own interests is known as psychological egoism (McConnell, 1978). Based on this theory, individuals take in community activities based on egoism. Taxpayers driven by personal interest tend to evade their tax liability.

#### 2.1.2. Theory of tax evasion

According to the theory of tax evasion, persons and businesses pay taxes by evading a specific portion of their taxes. Paying taxes is a forced behavior for them, because they believe that if they do not pay taxes, they will be punished by the state (Sandmo, 2005; López, 2017; Mannan et al., 2021). The theory of tax evasion indicates that taxpayers evade their income tax for their interest due to egoist behavior. If possible, the taxpayers intend to evade their taxes completely. The reason why they don’t do so is that the chance of not being discovered by the authorities is almost zero. Additionally, it was presumptive that the government used the taxes and fines collected from those tax evaders for the purposes having nothing to do with the taxpayers (D’Souza, 2016). Therefore, considering personal interests, when the expected income of tax evasion is greater than its cost, the taxpayers tend to evade their taxes.

The theory of psychological egoism and the theory of tax evasion have similar bases. Both are based on personal interest, affect tax revenue collection performance, and ultimately harm societal development. Almost all researchers agreed that those theories are centered on the egoist behavior of individualism.

### 2.2. Empirical review

Various researchers explain the main distinction between tax avoidance and tax evasion. One is legal, whereas the other is illegal (Adebisi and Gbegi, 2013; Frank and Angaye, 2020; Alstadsæter et al., 2022). The legal use of the tax system to one’s advantage to legally reduce the amount of tax payable is known as tax evasion. Conversely, tax evasion is an act of reducing taxes without authorization (Montenegro, 2021). The shadow economy is linked to tax fraud and avoidance, according to Choi and Park (2022). The shadow economy is also an economy where people hide their actual and taxable revenue from businesses and other lawful ventures to avoid paying taxes. The government has protested against these two misdeeds numerous times (Angell, 2018). However, some high-income companies and individuals use tax avoidance strategies to avoid or minimize taxes, or they purposefully use fictitious methods with the assistance of tax officials to evade the whole amount of tax.

Tax evasion is defined as the illegal use of the tax system to one’s advantage to lower the amount of tax using illegal means. Tax evasion also refers to any criminal endeavor to avoid paying taxes (Helhel and Ahmed, 2014). Tax evasion significantly impacted the total revenue in Kenya and Rwanda and affected the country’s development differently (Kamau et al., 2012; Amani and Harelimana, 2017). The above researchers and others assured that tax evasion is a hazardous activity that seriously harms society.

According to the research finding, people behaved to benefit their business in the Netherlands, France, and Belgium and compared people’s behavior and how they act self-centric behavior (Choi and Park, 2022; Flanders and Wallonia). The association between personal income tax evasion and cultural characteristics, such as religiosity, government trust, and legal enforcement in Nigeria is investigated (Weigel et al., 1987; Kaulu, 2022). In discussion, taxpayers hate tax evasion activities; however, they engage them in different practices.

The study found that trust in government and law enforcement significantly impacts tax revenue in Nigeria. However, there was no significant role in preventing tax evasion and avoidance. The study investigated the factors influencing tax evasion in Nigeria, and the results showed that the government and law enforcement were not doing their job properly (Modugu and Anyaduba, 2014). Meanwhile, in Ethiopia, taxpayers evade their taxable income by supporting fictitious documents, overstating their expenditures, and not using reliable invoices (Gashaw and Ayalsew, 2019; Manaye et al., 2020; Mengistu et al., 2022). The above research result assured that in Ethiopia, tax evasion is also a critical problem and needs special attention. Additionally, all researchers agreed that taxation has a negative effect on taxpayers’ selfish and egoist behaviors.

On the other hand, some tax experts in tax offices help taxpayers engage in tax evasion to receive benefits from them. Taxpayers develop egoist behavior by evading taxes and preparing falsified documents based on the direction of some corrupted tax experts. In that instance, tax evaders engaged in malpractices; the poor moral (selfish) person has a chance to take advantage of the egoists purposefully (Frecknall-Hughes et al., 2017; Tadesse, 2022). Taxpayers’ psychological egoism always stands for the benefit of self-interest. Under egoistic behavior, taxpayers prefer a low tax rate that minimizes their tax liability (Boadway et al., 2007; Kaulu, 2022). Taxpayer egoism tends to stand on the side of individualism and that may harm societies’ development. Therefore, it needs special attention to minimize tax evasion and psychological egoist practices.

According to recent research results, tax non-compliance has adverse effects on the capacity to generate the required amount of tax, especially in developing nations. The phenomena of tax education limitations, falsified taxpayers’ documents, and the inconvenience of paying taxes have become crucial to increase tax revenue performance (Trawule et al., 2022). Taxpayers’ lack of awareness, motivation, and tax-paying ability led to their anxiety and misunderstandings with revenue offices. This is because tax laws and procedures are complicated and confusing for taxpayers. Tax education work should be given properly, especially in developing countries, so that people can perform their duties properly. Tax compliance can be enhanced through better tax decisions, more transparent general tax laws, and incentives for compliant taxpayers (Boadway et al., 2007; Frecknall-Hughes et al., 2017).

Although the Amhara Regional Revenue Bureau has a legal structure to punish tax evaders, many questions have been raised about the proper implementation of the new tax laws and administration efficiency and effectiveness. Generally, tax law, administration, and tax information activities do not focus on specific taxpayers who repeatedly evade taxes, but on all taxpayers, which is not conducive to finding tax evaders. One of the main ways to solve the problem of tax evasion is to reduce the psychological egoism of taxpayers. Empirical evidence shows that tax evasion and the psychological egoism of taxpayers exist in various countries, especially in developing countries. It is necessary to comprehensively solve the problem of tax evasion and the psychological egoism of taxpayers.

### 2.3. Tax evasion, emotions, and tax compliance

#### 2.3.1. Tax evasion and emotions

Trust is good, but control is better. The advice for combating tax evasion is to deter illegal behavior with rigid audits and harsh fines. But control and punishment may have unintended side effects; therefore, psychological variables (e.g., attitudes toward taxation, social norms, and perceived fairness) are receiving increased attention (Bazart and Bonein, 2014; Coricelli et al., 2014; Kirchler et al., 2014; Barkworth and Murphy, 2015).

In addition, in most cases, the presence of sympathy encourages more tax compliance and decreases psychological egoism. Tax evasion emanates from noncompliant taxpayers’ behavior and influences the whole tax performance. Priming to elicit empathy improves tax compliance. These findings support the inclusion of noneconomic factors in tax compliance behavior analysis (Calvet, 2013; Olsen et al., 2018; Paleka et al., 2022).

Therefore, tax evasion practices are done with intentional and unintentional emotions, so it is better to strengthen citizens’ respect for the tax law and support tax authorities’ day-to-day activities. These practices contribute to an increase in compliant taxpayers. Tax evasion is minimized through continuous build-up mechanisms of taxpayer emotions, and all the findings of the above research agreed that tax evasion is highly and sustainably minimized by the development of positive emotions.

#### 2.3.2. Emotions and tax compliance

The enforcement capacity (i.e., high power) of tax authorities induces negative emotions while increasing enforced compliance and decreasing the willingness to evade. Trust, on the other hand, reduces negative emotions while increasing positive feelings, which are associated with voluntary compliance intentions. Furthermore, a combination of high power and high trust reduces negative feelings and increases compliance intentions while decreasing readiness for evasion (Muehlbacher and Kirchler, 2010; Olsen et al., 2018; Bruno, 2019).

Positive emotions and emotions related to feelings of self-blame have a similar impact in both Austria and Italy. Emotional experiences play an important role in tax compliance decisions. Thus, tax authorities need to take into consideration specific emotions elicited by different tax-related activities and interactions with the authorities (Alm and McClellan, 2012; Casal et al., 2016; Enachescu et al., 2019; Privitera et al., 2021).

Hence, based on the above literature, if taxpayers respect the tax laws either afraid of being caught and fined (enforced compliance) or feel obligated to contribute their fair share (voluntary cooperation), the psychological influence of the tax law is better. The above findings are also supported by a growing body of empirical research. The psychological approach to taxpayer emotions has influenced how tax authorities regulate citizen behavior.

### 2.4. Measurements of all variables

#### 2.4.1. Dependent variable

Tax revenue collection performance is the dependent variable in this study. As the percentage of GDP varies from nation to nation and from sub-region to sub-regions in Sub-Saharan African countries, the performance of tax revenue collection is measured as total tax revenue (Alabede, 2015; Adandohoin, 2018). Ineffective tax collection efforts on the part of the government or insufficient tax structure policies can lead to poor tax performance in producing revenue (Teera and Hudson, 2004; Bahl and Martinez-Vazquez, 2008). Tax changes may not have improved tax revenue collection due to factors including fiscal corruption, the damaging impact of war on the economy, abusive tax exemptions, and a lack of attention to expanding the tax base (Morrissey et al., 2016; Ndoricimpa, 2021). This research measured tax revenue collection performance based on the approved measuring tools of the above research. Three instruments were utilized to measure tax revenue collection performance (Mu et al., 2022). See Appendix A.

#### 2.4.2. Independent variables

Tax evasion, tax education, and technology are independent variables.

##### 2.4.2.1. Tax evasion

With the fully modified ordinary least squares method, the scope of tax evasion from 1980 to 2019 was estimated (FM-OLS). According to the findings, tax evasion accounts for 16.7 percent of Jordan’s tax collections, compared to the average rate of 2.7 percent of GDP. The GDP, tax receipts, public debt, public and private investment, and both public and private consumption were all influenced by tax evasion (Ghazo et al., 2021). The variable tax evasion was measured. Many questionnaires measure tax evasion, and this research takes the relevant questions from Mughal (2012), Folayan and Adeniyi (2018), and Nangih and Nkemakola (2018); the measurements are in Appendix A.

##### 2.4.2.2. Tax education

While tax education and the ease of filing taxes operate as grease in the wheels of committed tax compliance, fear-appealing messages act as sand in the wheels of committed tax compliance (Trawule et al., 2022). In Lagos State, Nigeria, a tax education program focused on informing taxpayers about the socioeconomic ramifications of tax evasion options, and the transparent and responsible use of tax revenues had a major impact on taxpayers’ voluntary compliance. On this premise, the study concluded that tax education should be available to students pursuing non-accounting courses and accounting professionals at all levels. To encourage voluntary compliance, the government and other stakeholders should collaborate to reduce tax complexity and compliance costs (Olowookere and Fasina, 2013).

The impact of tax education on tax evasion is significant. As a result, this research concentrated on taxpayers to investigate a targeted respondent. Even though tax education has been measured with various measuring tools, the findings from previous research (Olowookere and Fasina, 2013) were measured and adopted in this investigation. The contextualized instruments contained five construct measuring items to achieve the study’s aims. Tax education was computed and measured by the adopted measurement tools (Akintoye and Onuoha, 2019; Daniel and Esther, 2019; Onuoha et al., 2019), and three measuring instruments were used to measure the impact of tax education on tax revenue collection performance, and the measuring tools are in Appendix A.

##### 2.4.2.3. Technology

In leading industries nations, information technology has been embraced and enhanced tax administration. The previous study showed that the usage of information technology contributed to an improvement in tax administration in Nigeria by 76.3% (Adeyeye, 2019). The adoption of blockchain technology may significantly impact the national economy as well as the tax section in the VAT system (Eilu, 2018; Fudamu et al., 2019; Setyowati et al., 2020). The impacts of technology on tax revenue collection performance were measured through different measuring tools (Weigel et al., 1999; Korndörfer et al., 2014). Based on the above research findings, this research used three measuring instruments (questions) to manage the impacts of technology on tax revenue collection performance, see Appendix A.

#### 2.4.3. Measurements of mediator variable

Taxpayers’ psychological egoism is the mediator variable. The concept of egoism is discussed in Weigel et al. (1999). Tax evasion intents may be influenced by egoism, defined as the excessive concern with one’s advantage or pleasure at the price of the community’s welfare (Weigel et al., 1999). It is characterized as a person’s ongoing controversy for their benefit or pleasure at the price of the community’s well-being. Egoistic individuals are expected to lie if it benefits them; egoism may influence tax evasion (Korndörfer et al., 2014). In a tax evasion case, taxpayers would respond based on the amount of money they stand to lose, as suggested by the rational choice model (Murphy, 2004). As a result, egoism is a significant predictor of tax evasion intents. The impacts of Taxpayers’ psychological egoism were measured with the predefined measuring tools measured by research findings (Weigel et al., 1999; Korndörfer et al., 2014). Based on the above research, this research used four measuring instruments (questions) to examine the impacts of taxpayers’ psychological egoism on tax revenue collection performance. All the measuring tools are in Appendix A.

#### 2.4.4. The relationship between independent variables and revenue collection performance

To investigate the impact of the mediator variable of taxpayers’ psychological egoism, linked tax evasion and other related independent variables with tax revenue collection performance, the researcher employed previously validated measuring items (Kaulu, 2022). This study tested the impact of tax evasion, taxpayers’ psychological egoism, tax education, and technology on tax revenue collection performance, as well as the mediating effect of taxpayers’ psychological egoism.

##### 2.4.4.1. Tax evasion and revenue collection performance

Compared to developed countries, tax evasion practices in developing countries are worse. Because nations are powerless to stop it, tax evasion is like a pandemic for them. Tax evasion, estimated to cost 20% of income tax collection, negatively impacts governments’ ability to raise the standard of living for their inhabitants and allocate a budget for public expenditures. It has become a disease of the nation’s economy (Ameyaw et al., 2015; Palil et al., 2016). As if tax evasion is a pandemic in developing countries, it affects tax revenue performance alarmingly and needs special attention to stop early. So, tax evasion is a critical problem for revenue collection performance. The other researcher (Adebisi and Gbegi, 2013; Ogbueghu, 2016) expressed that the twin ideas of taxes have a detrimental effect on Nigeria’s economy, implying the possibility of reducing rather than eliminating tax avoidance and evasion. It was also discovered that tax evasion and avoidance negatively impacted revenue collection and contributed to a decline in the country’s government revenues, particularly at the state level. The revenue generation of any state depends on the amount of tax revenue generated in that given state. Tax evasion highly affects revenue generation tax evasion decreases revenue generation and vice versa (Levin and Widell, 2014; Raza and Naqvi, 2016; Dosumu et al., 2020).

Therefore, tax evasion and revenue collection performance are inversely proportional. All researchers agreed that tax evasion significantly and negatively affects revenue collection performance. In most cases, tax evasion is reflected in different ways, like corrupt practices, which are reflected in egoist behavior and self-centrism.

##### 2.4.4.2. Psychological egoism and revenue collection performance

Attitudes regarding tax collecting were determined by psychological egoism and value orientation. Psychological egoism and attitudes toward taxation, in general, are correlated. A considerable impact on revenue collection was confirmed by the expected interaction between psychological egoism and performance in revenue collection (Arrington and Reckers, 1985; Kirchler, 1997). On the contrary, even if psychological egoism negatively affects revenue performance, the amount of influence is not that much. Psychological egoism is directly or indirectly interlinked with tax evaders’ actions (Feld and Frey, 2007; Malezieux, 2017). The above researchers agreed on the negative impact of psychological egoism on revenue collection performance, whether the impact is significant or insignificant. Especially the first two researchers assured the impacts are significant and need great effort.

##### 2.4.4.3. Tax education and revenue collection performance

The research result indicates taxpayer education is a powerful instrument for increasing people’s willingness to voluntarily pay taxes, which is essential for generating the urgently required tax revenues to support the achievement of the Sustainable Development Goals of the country it is provided through in-depth, frequently ongoing interactions with various audiences through social media campaigns, tax fairs, and television programs. Tax education significantly and positively influences revenue collection performance (Mabonga et al., 2015; Mu et al., 2022). In addition, tax education creates awareness for taxpayers and the entire society, including students and other parts of the community. Tax education for citizens can strengthen the establishment of tax compliance. In Nigeria, tax education and enlightenment positively and significantly affected the total tax revenue. Radio and television were revealed as the strongest tax education channels (Akintoye and Onuoha, 2019; Khalil and Sidani, 2020). So, tax education in any circumstance plays an important role in increasing revenue collection performance. The above research agreed that tax education plays a vital role in revenue collection performance.

##### 2.4.4.4. Technology and revenue collection performance

Technology in revenue collection performance is the bloodstream of taxation. However, most developing countries were unable to pay close attention to advanced technology in taxation. In Korea, the inception of technology in taxation (electronic tax invoicing) is credited with reducing the cost of tax compliance and improving business transaction transparency. The country’s success with electronic tax invoicing resulted from effective policy design and implementation, significantly impacting revenue collection performance (Hyung Chul, 2016). Information technology significantly impacts revenue collection in Yola, Adamawa State. It was discovered that digital devices and Internet access were not kept up to date, and there is no web portal network for people to file their taxes online efficiently. When tax administrators pay attention to technology, revenue collection will significantly boost and vice versa (Allahverdi et al., 2017; Fudamu et al., 2019; Mary et al., 2020). The above literature about technology agrees that effective and efficient revenue collection depends on the priority set up of tax administration.

### 2.5. Hypothesis on the independent, the mediating, and the dependent variables

#### 2.5.1. Tax evasion

Tax evasion has a detrimental and severe effect on the performance of tax revenue collection. The phenomenon of tax evasion is one of the challenges that officials in charge of economic policies and tax administration, as well as their collectors, face, particularly when the state experiences low revenue and high spending bills while needing to provide infrastructure and public services to achieve economic development. Due to tax evasion or avoidance, national governments will encounter significant budget deficits if they cannot collect all of the anticipated tax revenues. To make up for this shortfall in the financing, these governments may decide to impose additional taxes, broaden their tax bases, or borrow more money (Gashaw and Ayalsew, 2019; Ghazo et al., 2021). Thus, hypothesis 1 is proposed as follows.

*Hypothesis 1 (H1)*: Tax Evasion has a negative and significant impact on TRP.

#### 2.5.2. Tax education

To meet tax compliance issues, efficient and effective tax education for SMEs in emerging nations like Tanzania is essential. Despite Tanzania’s trend toward tax changes since 1998, the impacts of tax education on Small and Medium Enterprises (SMEs) are still subpar, and many SMEs continue to view the taxing process and cost as obstacles to their success. Tax education positively and significantly impacts Tanzanian tax revenue collection performance (Onuoha et al., 2019; Ndubula and Matiku, 2021). In addition, tax education strengthens tax revenue collection performance since it is considered that tax non-compliance has adverse effects on the capacity to generate revenue. Especially in developing nations, tax education, fear-appealing, and the convenience of tax have become crucial in today’s tax revenue discourse (Trawule et al., 2022).

Therefore, taxpayer education can be a fundamental instrument to increase people’s willingness to pay taxes willingly, as well as play a crucial role in mobilizing the tax revenues that are urgently required to assist in accomplishing sustainable development goals of the country. So, hypothesis 2 is anticipated as follows.

*Hypothesis 2 (H2)*: Tax Education has a positive and significant impact on TRP.

#### 2.5.3. Technology

Tax authorities’ increased reliance improves the delivery of public services and financial efficiency on e-government-driven technologies, such as electronic tax filing. Several tax authorities worldwide are implementing electronic tax administration systems to communicate with the taxpaying public in tax collection, administration, and compliance settings to increase the effectiveness and efficiency of tax administration and collection. Technology has a vital role and positive impact on tax collection performance (Olatunji and Ayodele, 2017; Okunogbe and Pouliquen, 2018; Hamza et al., 2021).

Moreover, by establishing strong relationship mechanisms between the tax revenue authority and micro businesses and using digital technology solutions to tackle challenges, issues like poor record keeping, ignorance of tax payment procedures, unknown tax collection channels, and multiple taxes can be addressed. To address the issues, some digital technology solutions were provided, which led to the introduction of the practical component of tax administration that can serve as guidance for tax administrators and policymakers (Ajala and Adegbie, 2020; Oreku, 2021). Therefore, technology, especially electronic tax system, facilitates and supports tax administration and has a positive and significant impact on tax revenue collection performance. Thus, hypothesis 3 is proposed as follows.

*Hypothesis 3 (H3)*: Technology has a positive and significant impact on TRP.

#### 2.5.4. Taxpayers’ psychological egoism

Tax morals, complexity, knowledge, and awareness impact the purpose of collecting taxes, and taxpayers’ psychological egoism is a crucial factor. Policymakers must lessen tax complexity to reduce taxpayers’ psychological egoism. They need to raise standards of morality, make more tax information available, and raise tax literacy. These all need to take taxpayers’ psychological egoism into account. To ensure that patriotism and altruism (the antithesis of egoism) start early in life, educators, tax policymakers, and practitioners must instill moral values in children’s curricula. Taxpayers psychological egoism has a very unfavorable and significant impact on tax revenue collection performance (Weigel et al., 1999; Kaulu, 2022).

On the other hand, taxpayers’ psychological egoism only focuses on personal interest and affects community development plans, which erodes the community’s trustworthiness (Kirchler, 1997; Khurana and Diwan, 2014). Taxpayers’ psychological egoism significantly and negatively affects tax revenue collection performance. Therefore, hypothesis 4 is predictable as follows.

*Hypothesis 4 (H4)*: Taxpayers’ psychological egoism has a negative and significant impact on TRP.

### 2.6. Conceptual frame work of the research

**Figure 1:**
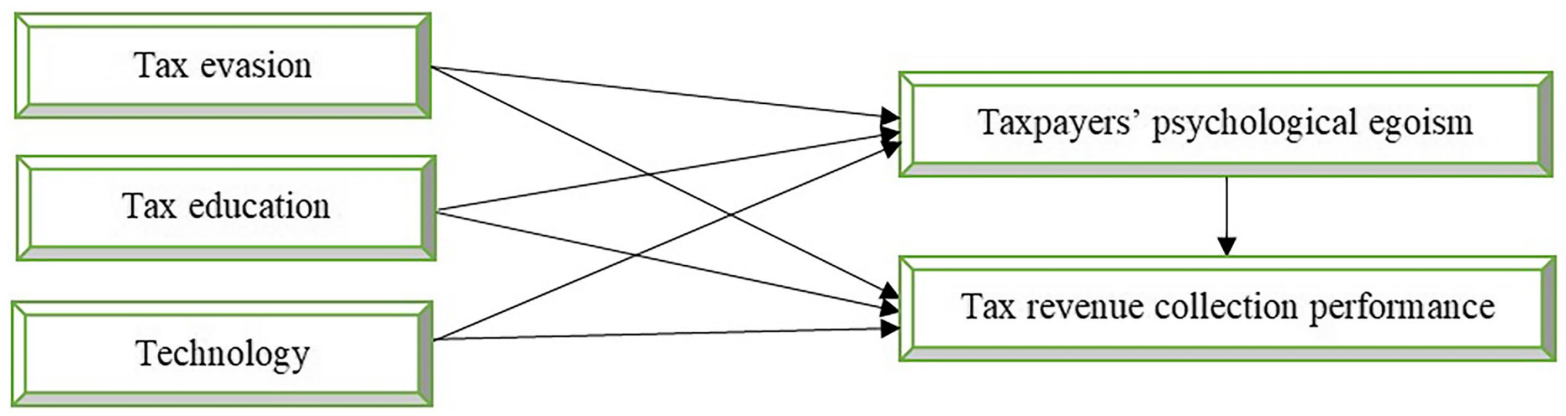
Conceptual framework.

The following conceptual research model, Figure 1, was developed to reveal the relationships among the independent, mediating, and dependent variables. Tax evasion, tax education, and technology are the independent variables. The mediating variable is taxpayers’ psychological egoism, and the dependent variable is tax revenue collection performance. Following that, the conceptual framework of the succeeding model aimed to validate, estimate the fitness, and support the stated hypotheses by employing the indirect and direct impacts of the mediation variable of taxpayers’ psychological egoism. As shown in Figure 1, tax evasion, tax education, and technology may not only directly impact tax revenue collection performance, but also indirectly affect tax revenue collection performance via taxpayers’ psychological egoism.

## 3. Research methodology

### 3.1. Research design

Research methodology is a strategy for methodically resolving a research issue. It is a science that examines the methods used in a scientific investigation (Kothari, 2004). This research used a survey research design because of limited resources. It also used simple random sampling techniques and quantitative analysis with both primary and secondary data sources. For the primary source, a closed-ended questionnaire was employed. The questionnaire had five stages Likert scale used for responses. Three hundred ninety-five (395) taxpayers of the Amhara Region were selected from a total of the taxpayer in the region. At the same time, the study also utilized secondary sources from published journals and books, tax proclamations, and other Internet sources. Structural Equation Modeling with AMOS software was applied to analyze data and explore linear causal relationships between variables.

### 3.2. Sample size and sample techniques

In the Amhara Region, there are three metropolitan and five regio-politan cities that can represent the region’s VAT-registered taxpayers which accounts for more than 65%. The researcher took the sample size from eight cities. This study tried to control the possible bias and sampling errors. The researchers selected individual taxpayers from the population of 29,540 VAT-registered taxpayers to collect the relevant data, and the determined sample size is 395. The sample was obtained using the probability of simple random sampling techniques. A sample size of 395 was calculated according to the Yamane formula determination of estimating sample size (Yamane, 1967).

Where:

*n* is the sample size,

*N* is the population size, and

*e* is the error term (0.05).


n=N/(1+N(e)∧2)

n=29,540/(1+29,540(0.05)∧2)

n=394.65≈395 respondents were selected based on the predefined criteria.

### 3.3. Data collection instrument and administration

This research focused on exploring the impacts of taxpayer egoism and tax evasion on tax revenue performance in the Amhara Region. Amhara Region is one of the nine regional states of Ethiopia with a population of over 20 million people, which is second to the Oromia Region of the country and has a large number of taxpayers, out of which 29,540 taxpayers are VAT registered. The study used simple random sampling techniques and randomly selected the respondents based on the value of the sample size.

A closed-ended questionnaire was prepared to collect the primary data. This research organized the data and collected data from VAT-registered taxpayers. The researcher designed this structured questionnaire to collect data on respondents’ thoughts on tax evasion, tax education, technology, taxpayers’ psychological egoism, and tax revenue collection performance. Ten undergraduate students distributed the questionnaires and collected the questionnaires with a continuous follow-up by the researcher, and all copies of the questionnaire were successfully returned. The researcher coded and entered the data into the SPSS software, administered it based on the appropriate software, and analyzed the result. This questionnaire was adapted based on previous studies (Harelimana, 2018; Akintoye and Onuoha, 2019; Onuoha et al., 2019; Night and Bananuka, 2020; Le et al., 2021; Kaulu, 2022; Mu et al., 2022) to measure the impacts of tax evasion, taxpayers’ psychological egoism, and technology on tax revenue collection performance.

### 3.4. Methods of data analysis

This research used descriptive statistics to analyze the respondents’ background information and summaries data samples in the Amhara Region, Ethiopia. The respondents’ distribution (gender, age, marital status, educational qualifications, and work experience) was presented using frequencies and percentages as part of the descriptive analysis. For each statement, frequencies, means, and SD were used to evaluate the replies. The structural equation model and multiple regression analysis based on the softwares of SPSS and AMOS were utilized for data analysis to test the hypotheses.

### 3.5. Model specification

The variables in this research were guided by a statistical model whose relational functions explained the model’s role. The information was gathered to clarify the impact of tax evasion, tax education, technology, and taxpayers’ psychological egoism on tax revenue collection performance. The following regression model was developed based on the variable measurements to guide the examination of the variables’ effects and to indicate the relationships that existed between them:


Y=F(X)M

Where: Y = tax revenue collection performance, the dependent variable; X_1,2,3_ = tax evasion, tax education, and technology, the independent variable; M = taxpayers’ psychological egoism, the mediating variable.

The model is then rearranged for regression analysis as:


$$\mathrm{TRP}=\beta_{0}+\beta_{1}\mathrm{TAEV}+\beta_{2}\mathrm{TAED}+\beta_{3}\mathrm{TECH}+\beta_{4}\mathrm{EGO}+\varepsilon$$

Where TRP = tax revenue collection performance, the dependent variable; TAEV=tax evasion, TAED=tax education, and TECH=technology, the independent variables; EGO=taxpayers’ psychological egoism, the mediating variable, β_0_ is a constant term; β_1,2,3,4_ is the parameter estimation coefficient of tax evasion, tax education, technology, and taxpayers’ psychological egoism; ε is the error term.

## 4. Data presentation, analysis, and interpretation

The researcher selected Structural Equation Modeling (SEM) to test the hypotheses (Byrne, 2001; Markus, 2012; El-Sheikh et al., 2017). The mediating variable taxpayers’ psychological egoism should be analyzed using SEM because of the similar and related nature of the variables, and model fitness is also part of the measurement tools. It measures and analyzes the relationships between observed and latent variables.

### 4.1. Demographic data analysis

According to the demographic data of 395 sample respondents, males made up 74.48 percent (294), and females made up 25.52 percent (101) of the total. The result indicates that female participation needs particular affirmative action. Most respondents (40%) were between the ages of 21–30; 28% were between the ages of 31–40; 18% were between the ages of 41–50; 14% were beyond 50 years, and 20 years of age and below were zero. Based on the age category results, the young taxpayers participated more actively. The majority of respondents (46.2%) were with a diploma, 21.4% were in high school, and 20.3% were undergraduates (degree holders), followed by 12.1% with postgraduate degrees. This suggests that most respondents were in diploma and could supply the study with valuable information.

### 4.2. Validity and reliability test

Based on Fornell and Larcker (1981), the Average Variance Extracted (AVE) higher than 0.5 is acceptable, and according to Hair et al. (2017), Cronbach’s Alpha ≥0.7 is in a good range. Because AVE > 0.5, the computation indicates that all model variables are believed to meet the criterion for discriminant validity. Additionally, all Cronbach’s Alpha is higher than 0.5, demonstrating the great dependability and compliance of all the constructs on the calculated model. The results of the reliability and validity tests are summarized in Table 1 below.

**Table 1:** Results of reliability and validity tests.

Constructs	Items	Factor loading	Chronbach’s alpha	Composite reliability (CR)	Average variance extracted (AVE)
Tax evasion	TAEV1	0.582	0.901	0.484	2.546
	TAEV2	0.812			
	TAEV3	0.675			
Tax education	TAED1	0.884	0.861	0.621	2.134
	TAED2	0.625			
	TAED3	0.833			
Technology	TECH1	0.754	0.891	0.551	2.344
	TECH2	0.586			
	TECH3	0.862			
Taxpayer egoism	EGO1	0.808	0.871	0.507	2.9685
	EGO2	0.724			
	EGO3	0.684			
	EGO4	0.622			
Tax revenue collection performance	TRP1	0.579	0.868	0.401	3.393
	TRP2	0.611			
	TRP3	0.702			
	TRP4	0.673			

TAEV, tax evasion; TAED, tax education; TECH, technology; EGO, taxpayers’ psychological egoism; and TRP, tax revenue collection performance.

### 4.3. Correlation matrix and discriminant validity

The covariance values show a strong relationship between the dependent variable, tax revenue collection performance, and the independent variables, tax evasion, tax education, and technology, and the mediating variable, taxpayers’ psychological egoism. At the 5% significance level, the square root of the average variance extracted (AVE) from the observed variables suggests that all independent and dependent variables have a positive and significant relationship. Furthermore, the outcome variable, tax revenue collection performance, is positively and significantly related to tax evasion, tax education, and technology. As shown in Table 2, the mediation variable, taxpayers’ psychological egoism, also supports the interconnections between covariates.

**Table 2:** Correlation matrix results.

	TRP	TAEV	TAED	TECH	EGO
TRP	1				
TAEV	0.627^**^	1			
TAED	0.718^**^	0.598^**^	1		
TECH	0.752^**^	0.615^**^	0.579^**^	1	
EGO	0.638^**^	0.568^**^	0.663^**^	0.557^**^	1

TAEV, tax evasion; TAED, tax education; TECH, technology; EGO, taxpayers’ psychological egoism; and TRP, tax revenue collection performance; ^**^means the results are significant at the 5% significance level.

### 4.4. Multiple regression analysis

Multiple regression analysis can be used to identify the interaction degree between variables (Alexopoulos, 2010). This research considered tax evasion, tax education, and technology as independent variables, taxpayers’ psychological egoism as a mediating variable, and tax revenue collection performance as a dependent variable. Tax evasion (TAEV) was determined to have a value of 0.774, tax education (TAED) of 0.693, technology (TECH) of 0.878, and taxpayers’ psychological egoism (EGO) of 0.893, in the direct impact from the independent to the dependent variable. The weighted multiple regression analysis results show that all regression outcomes have positive and significant correlations with tax revenue collection performance.

Contrarily, the indirect effects of the independent variable on the dependent variable through the mediating variable had parameter estimates (β) values of tax evasion (TAEV) 0.875, tax education (TAED) 0.883, and technology (TECH) 0.736. The critical ratio (C.R) weighted score was higher than 1.96 when the parameter estimations (β) were compared to their pertinent standard error (S.E), with a 0.05 value of p suggesting positive and significant integration, as shown in Table 3.

**Table 3:** Regression weights for the level of significant and critical ratio.

Latent variable	Path	Measurement variables	Standard estimate (β)	S.E.	C.R.	Sig.
TRP	**←**	Tax evasion	0.774	0.082	8.34	0.000^***^
TRP	**←**	Tax education	0.693	0.197	10.34	0.000^***^
TRP	**←**	Technology	0.878	0.097	4.62	0.000^***^
TRP	**←**	Taxpayers’ psychological egoism	0.893	0.128	10.57	0.000^***^
EGO	**←**	Tax evasion	0.875	0.079	9.83	0.000^***^
EGO	**←**	Tax education	0.883	0.094	12.81	0.000^***^
EGO	**←**	Technology	0.736	0.168	11.16	0.000^***^

TAEV, tax evasion; TAED, tax education; TECH, technology; EGO, taxpayer egoism; TRP, tax revenue collection performance SE, standard error; CR, critical ratio; and Sig, significant level; ^***^means the results are significant at the 1% significance level.

### 4.5. Testing the mediation role of taxpayers’ psychological egoism

The importance of a mediated effect can be determined using an estimate of its standard error. The Sobel test is the most common multivariate delta technique product of the coefficient test. The *z*-score of the mediated effect, on the other hand, is the mediated effect divided by its standard error. If the *z*-score is more than 1.96, it is a positive indication. Confidence intervals around the mediated effect can be calculated using the standard error.

Although there are other techniques for evaluating mediation, the distribution of the product (bootstrapping) approach is recommended for better type I error and performance (MacKinnon et al., 2004). As a result, this method was used to investigate the mediating role of taxpayers’ psychological egoism in the interaction between tax evasion, tax education, and technology. The significance of the indirect effect is the only criterion for mediation in this approach (Hadi and Abdullah, 2016; Hair et al., 2017; Meuleman, 2019). The estimated weight of the indirect effect is significant at the 1% significance level, according to the bootstrapping results.

Additionally, as indicated in Table 4, the results of the Sobel test and the mediation effects corroborate the dual effect (direct and indirect effect). It denotes that the relationship between the independent variable and the tax revenue collection performance is reliably mediated by taxpayers’ psychological egoism.

**Table 4:** The result of the mediation effect.

Mediation effects	Coefficient	Standard error	Soble test (*z*-score)	*p* value
TAEV**→** EGO**→** TRP	0.857	0.079	5.903	0.000
	0.893	0.128		
TAED**→** EGO**→** TRP	0.883	0.094	5.601	0.003
	0.893	0.128		
TECH**→** EGO**→** TRP	0.736	0.168	3.710	0.001
	0.893	0.128		

TAEV, tax evasion; TAED, tax education; TECH, technology; EGO, taxpayers’ psychological egoism; TRP, tax revenue collection performance.

### 4.6. Growth path modeling result analysis and the structural equation model

#### 4.6.1. Goodness-of-fit indices for structural equation models

The research chooses SEM to explore linear causal relationships between variables and simultaneously takes measurement error into account, making it more effective than regression studies. This research used SEM with AMOS software to analyze and cross-check the Chi-square (χ^2^), Root Mean Square Error of Approximation (RMSEA), and Goodness of Fit Index. SEM (structural equation modeling) is a commonly utilized research approach for verifying complicated phenomena. It is an equation system that formalizes the structural relationships between cause and effect variables in the area of interest. SEM is a data analysis approach frequently utilized in various fields due to its numerous benefits: For instance, measurement mistakes can be managed. Second, mediating variables are simple to apply. Third, the theoretical model can be statistically evaluated (Kang and Ahn, 2021).

According to statistics, (1) Chi-square (χ^2^) should have a non-significance (*p* > 0.05) according to the statistics, (2) Goodness of Fit Index (GFI) > 0.90, and (3) Root Mean Square Error of Approximation (RMSEA <0.08) are used in SEM to determine the adequacy and fittingness of sample size (Yay, 2017). The accepted Goodness of Fit Values is *x*^2^/d.f <3.0, 0.95 < CFI < 0.97; 0.85 < AGFI <0.90; 0.90 < GFI < 0.95; 0.90 < NFI < 0.95; and 0.05 < RMSEA < 0.08 (Hair et al., 2017). Table 5 shows the results of the Goodness of Fit Indices,which are within the acceptable ranges.

**Table 5:** Model fit indices.

Model	*x* ^2^	d.f	x^2^/d.f	GFI	AGFI	NFI	IFN	TLI	CFI	RMSEA
Measurement model	268.41	179	1.49	0.942	0.927	0.933	0.954	0.962	0.938	0.071
Recommended values		>0.0	<3.0	≥0.90	≥0.90	>0.90	>0.90	≥0.90	≥0.90	<0.08

*x*^2^, chi-square; d.f, degree of freedom; GFI, goodness of fit index; AGFI, adjusted goodness of fit index; NFI, normal fit index; IFN, incremental fit index; TLI, Turker–Lewis index; CFI, comparative fit index; and RMSEA, root mean square error of approximation.

#### 4.6.2. Result analysis for growth path modeling

**Figure 2:**
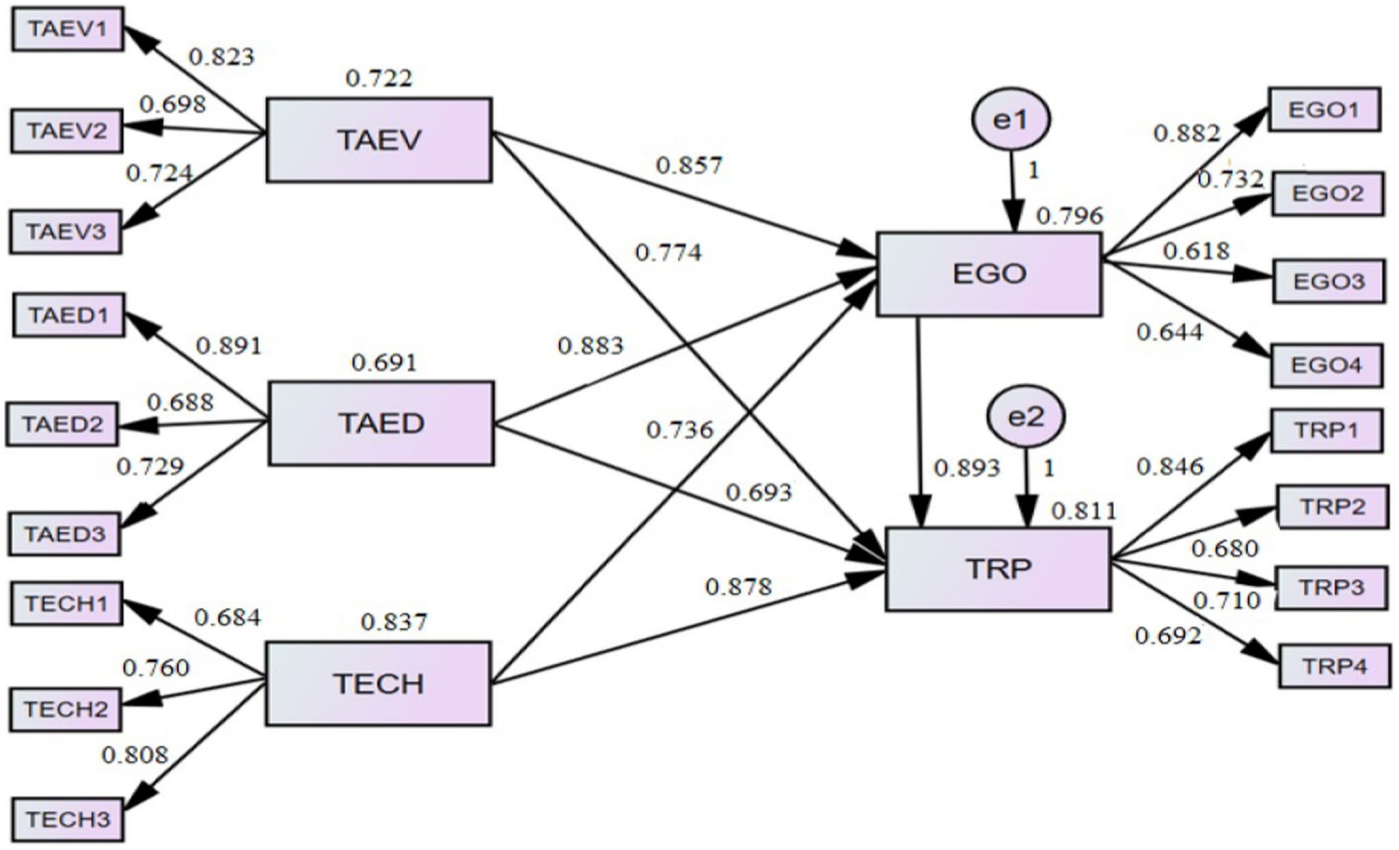
Conceptual framework path diagram.

According to the results of the direct path diagram modeling in Figure 2, tax evasion (β = 0.774^***^), tax education (β = 0.693^***^), and technology (β = 0.878^***^) all positively and significantly affect the dependent variable, tax revenue collection performance. Furthermore, the indirect path diagram modeling results show that tax evasion (β = 0.857^***^), tax education (β = 0.883^***^), and technology (β = 0.736^***^) all have a significantly positive impact on taxpayers’ psychological egoism. Meanwhile, taxpayers’ psychological egoism has a significantly positive impact on tax revenue collection performance (β = 0.893^***^).

### 4.7. Test of hypothesis and decisions

This research determines whether to reject or accept the alternative hypothesis based on the statistical test results. The researcher also explained the study assumptions based on the available effects and analyzed the data according to the other researchers’ findings. As a result, based on previous research, this study produced four potential assumptions that can be accepted or rejected based on research findings. This study aimed to fill in specific gaps in this area by offering hypothetical conclusions on how tax evasion and other related variables affect tax revenue collection performance in Amhara Region.

The hypothesis is accepted if a value of *p* < 0.05 exists between the explanatory and outcome variables (Dahiru, 2008). Based on Table 6, tax evasion (β = 0.774, *p <* 0.05), tax education (β = 0.693, *p <* 0.05), technology (β = 0.878, *p <* 0.05), and psychological egoism (β = 0.893, *p <* 0.05) all have significant impacts on tax revenue collection performance. Tax evasion and psychological egoism negatively affected tax revenue collection performance. On the contrary, tax education and technology positively affected tax revenue collection performance.

**Table 6:** Hypothesis testing results and decisions.

Hypothesis	Predicted hypothesis	Direction and structural path	Factor loading (B)	*p* value	Decision
Hypothesis 1	H1(−)	TAEV → TRP	0.774	(0.000)	Accepted
Hypothesis 2	H2(+)	TAED → TRP	0.693	(0.001)	Accepted
Hypothesis 3	H3(+)	TECH → TRP	0.878	(0.000)	Accepted
Hypothesis 4	H4(−)	EGO → TRP	0.893	(0.000)	Accepted

According to Table 6, all the hypotheses in this study are verified. As tax evasion significantly and negatively affects tax revenue collection performance, the hypothesis 1 is confirmed. Tax education significantly and positively affects tax revenue collection performance, so the hypothesis 2 is accepted. The technology significantly and positively impacts tax revenue collection performance, so hypothesis 3 is verified. Finally, taxpayers’ psychological egoism mediates significantly and negatively affects tax revenue collection performance, so the hypothesis 4 is confirmed. Overall, tax evasion, tax education, technology, and taxpayer egoism all play an important role in tax revenue collection performance.

## 5. Discussion

In previous studies in the Amhara Region, most of the time in the survey study, male participants take a higher share; however, in the case of the age difference, old aged and illiterates take more percent (Belay and Viswanadham, 2016; Tehulu, 2019). Similarly, this research result indicates that male respondents take the lion’s share and need particular affirmative action for female participants. But, young taxpayers with diplomas were more sound than others, indicating that it is promising for the future.

Tax evasion is negatively related to tax revenue performance, while tax education and invoicing are positively related to tax revenue performance (Kemme et al., 2020; Ndubula and Matiku, 2021; Mu et al., 2022). In the same result, this research assured that there is a negative relationship between tax evasion and tax revenue collection performance, and tax evasion happens in reporting and financial statement preparation. Taxpayers overstate their expenditures and understate their income. Because of the high-level psychological egoism of taxpayers, taxpayers evade vast amounts of tax. In these activities, taxpayer accountants support and show the loopholes in tax laws to the taxpayers, facilitating this misconduct. So, tax evasion alarmingly affects the total tax revenue and harms the Amhara Region economy.

Furthermore, the findings revealed a positive relationship between tax education and tax revenue collection performance; when the tax authority pays special attention to tax education, taxpayers are willing to pay their taxes on time and within the allotted time. On the contrary, if tax leaders give their focus only on collection activities, the taxpayers’ willingness to pay their taxes will decrease. Therefore, effective tax education activities is conducive to improving tax revenue collection performance and ultimately promoting the economic development of the region.

Researchers give empirical support for these research findings by demonstrating that tax evasion has a detrimental impact on tax revenue collection performance (Enofe et al., 2020; Frank and Angaye, 2020). The impact of technology on tax revenue collection performance in Southwest Nigeria had a statistically significant positive effect on tax revenue collection performance. Technology has resulted in lower tax collection performance due to a lack of updated and advanced technology (Frank and Angaye, 2020). Similarly, this research finding revealed that tax evasion negatively affects tax revenue collection performance, whereas technology positively impacts tax revenue collection performance.

The path diagram’s depiction of the indirect and direct relationship between the predictor variable and the outcome variable shows that, taxpayers’ psychological egoism has a significantly negative impact on tax revenue collection performance. Meanwhile, it serves as a vital mediator between other variables and tax revenue collection performance. Tax evasion, tax education, and technology can not only directly impact tax revenue collection performance, but also indirectly affect tax revenue collection performance via taxpayers’ psychological egoism.

## 6. Conclusion and recommendations

### 6.1. Conclusion

Tax evasion is the criminal act of underreporting income, overstating expenses, deductions, or exclusions to reduce accruing taxes or completely avoid paying them (Mughal, 2012; Adebisi and Gbegi, 2013). Using the structural equation model and multiple regression analysis, this research investigated how tax evasion, taxpayers’ psychological egoism, and other relevant factors like tax education, and technology affect tax revenue collection performance in the Amhara Region. The results reveal that tax evasion and taxpayers’ psychological egoism negatively affect tax revenue collection performance. Tax education and technology significantly and positively affected tax revenue collection performance. Moreover, taxpayers’ psychological egoism has a significant mediating effect on the relationship between tax evasion and tax revenue collection performance, as well as the impacts of tax education and technology on tax revenue collection performance.

In the Amhara Region, tax revenue collection performance has been understated due to improper tax administration, assessment, and collection. This could be because individuals and businesses habitually evade, and avoid taxes due to corrupt activities and numerous tax loopholes. The accomplishment or failure of any tax system is determined by how well it is administered and how well the tax law is interpreted and applied (Akintoye and Onuoha, 2019). Taxpayers psychological egoism affects the Amhara Region tax revenue collection performance hazardously. Most taxpayers depend on their interests and evade their taxes by falsifying their tax reports. The system of tax education is not appropriate and smart; due to these acts, taxpayers evade taxes intentionally by focusing only on their income. The existing technology is outdated and has served for the last 15 years, so tax auditors and tax intelligence could not find corrupt practices. This study’s findings align with those of Oseni (2017), Amah and Nwaiwu 2018, and Onwelumadu and Onuora (2021). In addition, tax evasion is a severe problem in Amhara Region, Ethiopia. It stems from various factors, including a lack of tax education and the absence of machine learning technology to estimate taxpayers’ actual taxable income. Influential leaders do not actively participate in tax education activities. In elementary education, students are not focus-areas on influencing their parents’ egoist behavior. The regional and country’s media do not prioritize tackling tax evasion, and egoist conducts. Due to the current security problems in the Amhara Region, most compliant taxpayers have developed egoist behavior psychologically and focused on their interests. Lack of understanding of the scope of tax laws and a lack of trust in the government’s ability to use tax revenue effectively make evasion very critical. When taxpayers’ psychological egoist behavior increases, tax revenue performance decreases. So, it is important to take comprehensive measures to reduce tax evasion and egoism to increase compliance taxpayers.

### 6.2. Recommendations

First, the tax system’s primary goal should be to promote economic development. Therefore, the tax system should promote economic progress rather than stifle it. The research suggests the government in Amhara Region formulate related financial and taxation policies that are matched to its economic growth goals.

Second, this study recommends that the system of tax education should include influential social leaders and political leaders who prioritize tax education activities. Tax education should activate based on the situation and start from elementary education, and the student can influence their parents’ egoist behavior. The media should identify and expose taxpayers’ psychological egoist actions to reduce such improper conduct.

Last, to reduce the tax evasion behavior resulting from taxpayers’ psychological egoism, the Amhara Region should implement the latest technology like developed countries. Artificial intelligence and machine learning technology can help decrease tax evasion misconduct. So, the Amhara region should be focused on these new strategies. Taxpayer egoism can be minimized by creating awareness about thinking for the next generation’s development.

## Data availability statement

The raw data supporting the conclusions of this article will be made available by the authors, without undue reservation.

## Ethics statement

Ethical review and approval were not required for the study on human participants in accordance with the local legislation and institutional requirements. Written informed consent from the participants was not required to participate in this study in accordance with the national legislation and the institutional requirements.

## Author contributions

RM, NMF, and LZ: conceptualization, methodology, and formal analysis. NMF: validation. NMF: investigation and writing original draft preparation. NMF and LZ: writing review and editing. RM and NMF: supervision. All authors contributed to the article and approved the submitted version.

## Conflict of interest

The authors declare that the research was conducted in the absence of any commercial or financial relationships that could be construed as a potential conflict of interest.

## Publisher’s note

All claims expressed in this article are solely those of the authors and do not necessarily represent those of their affiliated organizations, or those of the publisher, the editors and the reviewers. Any product that may be evaluated in this article, or claim that may be made by its manufacturer, is not guaranteed or endorsed by the publisher.

## Figures and Tables

**Table:** 

Variables	Items	Sources
Tax evasion	1. Cheating on tax payments is OK because government spending is not reasonably implemented.	Kaulu (2022)
	2. Taxpayers evade taxes because the tax system is unfair.	
	3. The controlling mechanism of tax evasion is weak and inefficient.	
Tax education	1. I generally receive sufficient information about my tax issues.	Akintoye and Onuoha (2019), and Onuoha et al. (2019)
	2. Tax education proactively encourage voluntary tax compliance	
	3. Taxpayers’ education programs reduce errors by empowering taxpayers with tax knowledge.	
Technology	1. Using Technology increased revenue collection.	Night and Bananuka (2020), and Le et al. (2021)
	2. Technology can support to struggle tax evasion and fraudulent activities.	
	3. Using new technology in tax system improves service delivery.	
Taxpayers’ psychological egoism	1. The egoist persons are evading taxes because of lack of tax education and transparency from government body	Kaulu (2022)
	2. A taxpayer should obey tax regulations to implement public infrastructure.	
	3. There is no technology to handle tax evaders that is why tax payers being egoist.	
	4. The biggest difference between most criminals and other people is that criminals are imprudent enough to get caught.	
Tax revenue collection performance	1. Amhara revenue authority has the capacity to identify tax evaders through audit.	Harelimana (2018), and Mu et al. (2022)
	2. Audits are conducted on a timely basis to verify if the taxpayer has correctly reported and assessed their obligations.	
	3. Effectively collect tax revenue and increase community participation in struggling tax evasion and fraudulent acts.	
	4. Public trust can emanate from showing construction of infrastructure to the public through the collected taxes.	

## Appendix

Appendix A Variables measurement tools.
